# Fungoid Pulp

**Published:** 1900-11

**Authors:** Walter W. Barton


### FUNGOID PULP.

BY WALTER W. BARTON, D.D.S.

On January 10 John D., aged eleven years, presented himself in the clinic for the purpose of having his upper anterior teeth regulated. Upon examination it was found that all of his six-year molars were badly decayed. The upper left first molar had a large cavity on the morsal surface which was almost completely filled with a red, spongy tissue, which had no connection to the lateral walls of the tooth, and was diagnosed as fungoid pulp. This was caused by exposure of the pulp by caries, and followed by continued irritation, which produced the low form of granulation tissue peculiar to this growth. This was somewhat sensitive, but not to the same degree as a healthy pulp.

The prognosis being favorable, the cavity was washed with a three-per-cent. solution of hydrogen dioxide and crystals of iodine packed around the bulbous mass, but could not be sealed in. The case was treated twice each week, and at the second sitting the pulp was greatly reduced in size. After applying the dam the cavity was again washed with hydrogen dioxide, and iodine crystals were sealed in with gutta-percha and gentle pressure used.

This treatment gave no pain, and at the next sitting all the bulbous portion of the pulp was absorbed. This was followed by two applications of carbolic acid and iodoform, when the pulp-canals were entirely cleansed and a dressing of iodoform and carbolic acid was placed in the canals for a few days. There being no putrescent odor at the end of a week, the canals, which were not closed at the apex, were filled with gutta-percha, the tooth was lined with oxyphosphate, and the remainder of the cavity filled with amalgam.

This treatment extended over a period of three weeks.

---

